# Changes in couple, parenting, and individual functioning following Family Expectations program participation

**DOI:** 10.1111/jmft.12613

**Published:** 2022-09-24

**Authors:** Allen W. Barton, Qiujie Gong, Scott M. Stanley, Galena K. Rhoades

**Affiliations:** ^1^ Department of Human Development and Family Studies University of Illinois at Urbana‐Champaign Urbana Illinois USA; ^2^ Department of Psychology University of Denver Denver Colorado USA

**Keywords:** couples, family expectations, marriage, parenting, program, relationship education

## Abstract

The current study investigated changes in couple, parenting, and individual functioning following participation in Family Expectations, a relationship and parenting education program for new or expectant parents. The sample comprised 339 couples who participated in most sessions of the Family Expectations program and completed assessments at three different time points over a 12‐month period. Study analyses examined: (a) change shortly following completion of the program, (b) associations between short‐term change and subsequent change in outcomes at 12‐month follow‐up, and (c) differences in short‐term change between married and unmarried couples. Significant improvements were observed in all three domains at short‐term follow‐up. Short‐term changes, particularly for psychological distress, were predictive of long‐term change in multiple domains. Few moderation effects by marital status were evident; those that appeared suggested stronger effects for married participants compared to unmarried. Study findings inform ongoing discussions into the utility of federally‐funded relationship and parenting programming.

In the early 2000s, the United States federal government began a funding program designed to support efforts to foster healthy couple relationships (Herman‐Stahl et al., 2021). Under the direction of the Administration for Children and Families (ACF), this initiative has resulted in a substantial increase in the availability of relationship education services to couples throughout the country, particularly those that are more socioeconomically disadvantaged (Hawkins, 2019). The initiative has also been accompanied with a corresponding critique regarding the appropriateness of such efforts (e.g., Karney et al., 2018). Although clear differences of opinion exist between advocates and proponents in this debate (Hawkins et al., 2013; Johnson, 2012), one common area of agreement from both sides is the need for increased empirical studies on these programs to better understand fundamental questions into the nature of change in couples following program participation.

The present study was designed to inform this need. Specifically, from a subsample of 339 couples who participated in an ACF‐funded relationship and parenting education program and provided responses at three time points, we investigate short‐term change in couple, parenting, and individual outcomes as well as the degree to which short‐term change predicted change at 1‐year follow‐up. Differences by couples′ relationship status (i.e., married vs. unmarried) were also investigated to consider whether certain types of relationships demonstrated greater change following program participation.

## FURTHERING RESEARCH ON RELATIONSHIP EDUCATION PROGRAMMING

Since its inception, this ACF initiative has funded program with various delivery approaches, including group‐based workshops, online programming, and community advertising. Equally diverse are the targeted audiences for these programs, which have included low‐income married couples, unmarried couples expecting a child, individual adults, and youth at the onset of romantic relationship formation (Herman‐Stahl et al., 2021). The current study considers change among participants in an ACF‐funded study of the Family Expectations program, a 12‐week group‐based in‐person program for expectant or new parents designed to provide couples with information and skills to help during this transition, including topics related to newborn care, stress management, coparenting, and couple functioning. Previous findings from this randomized controlled trial of the program indicated that couples assigned to Family Expectations demonstrated less destructive communication at 1‐year follow‐up compared to couples in the control condition (Ritchie et al., 2022). Further, some program effects were moderated by socioeconomic factors, such that more socioeconomically disadvantaged individuals demonstrated increased benefits in relationship communication and stability (ibid). No findings regarding short‐term program efficacy were permissible as control participants only completed a single follow‐up assessment 1 year from baseline.

The current study extends prior findings in three important ways. First, we investigate the degree of short‐term change after completing the Family Expectations program and examine how these changes are associated with longer‐term change. Investigation at multiple time points is important to consider time windows of change following participation as well as potential pathways of change. The inclusion of time points closer to completion of the actual program permits identification of processes that appear malleable to the intervention, with long‐term assessments helping to facilitate an understanding of durability of effects. Prior analyses of ACF‐funded programming include short‐ and long‐term evaluations (Doss et al., 2020; Rhoades, 2015; Hawkins et al., in press); the largest randomized trials commonly only included long‐term follow‐up (e.g., 12–15 months, 36 months; Lundquist et al., 2014; Moore et al., 2018; Wood et al., 2014), thereby precluding the ability to examine change closer to the end of couples' completion of the program.

As a second area of contribution, we investigated change with respect to couple, parenting, and individual outcomes. Although there is growing support for couple‐focused programming to elicit direct or indirect effects on parenting and individual outcomes (Barton et al., 2021; Lavner et al., 2020), such effects remain understudied and, with respect to the Family Expectations program, nearly nonexistent in the empirical literature. For nearly a decade, family scholars in this area have noted the need for greater research on participants' individual and parenting outcomes, particularly given the benefits to children that were one of the motivating reasons of this federal initiative to enhance couples' functioning (Cowan & Cowan, 2014).

Third, the current study investigates the degree to which relationship status moderates postprogram changes. Historically, most programs designed for expectant couples assumed that partners had previously committed to having a long‐term relationship together. Given societal shifts in nonmarital childbearing and relationship formation processes in general (Cherlin, 2010, 2014; Halpern‐Meekin, 2019), this assumption is less certain and there now exists substantially variability in participating couples' relationship stage (e.g., casually dating, highly‐committed dating, cohabiting, engaged, married). Despite the increased diversity of couple types seeking relationship education, few studies have included both married and unmarried couples and investigated if marital status differentially affected program‐related change. As one exception, Barton et al. (2018) found no differences in program effects between married and no‐married couples participating in the Protecting Strong African American Families program. Meta‐analytical findings of couple and relationship education programming more broadly suggest programs that involve samples of married couples (Hawkins & Erickson, 2015) or married and unmarried couples (Hawkins et. al., in press) report larger effect sizes with respect to couple outcomes compared to programs that targeted exclusively unmarried participants.

This variability in relationship status introduces a variety of complexities into relationship education services and questions as to whether certain relationship types will benefit more from participation than other types. One possibility is that unmarried couples—who, on average, are more at‐risk for relationship distress and dissolution than married couples (Guzzo, 2009; Manning et al., 2004)—will demonstrate greater improvements, consistent with research on the increased benefit of prevention programming for more at‐risk couples (Amato, 2014; Stanley et al., 2020). Conversely, a different line of thought suggests that married individuals will demonstrate more improvement from relationship education programming, at least with respect to couple outcomes. At the risk of stating the obvious, relationship education programs are designed to help people strengthen their relationship. Although each marriage is unique (in its strengths and challenges), every married individual is alike in having made an intentional decision and declaration of their commitment to this relationship (Stanley et al., 2010). Consequently, an individual engaging in efforts to strengthen this relationship is consistent with the previous (and perhaps current) convictions of wanting to be in this relationship for the long‐term and thus will exhibit greater improvement. A third possibility is that couples will benefit equally across relationship type.

In summary, the current study was organized around the following three questions: (1) do couples experience short‐term change in couple, parenting, and individual domains following participation in the Family Expectations program?, (2) is short‐term change associated with change at 12‐month follow‐up?, and (3) does marital status moderate short‐term change in outcomes?

## METHOD

### Participants and procedures

Participant enrollment spanned 2017–2019. Couples were recruited using radio advertisements, referrals from community organizations, and referrals from previous participants. Although there was no income‐based or socioeconomic requirement for eligibility, recruitment efforts targeted agencies that provide support to socioeconomically disadvantaged individuals and families. To be eligible, individuals had to be 18 years of age or older, in a committed romantic relationship, expecting a baby or had a baby within the past 3 months, the biological parents to that baby, able to participate in English, and have both partners willing to participate in the Family Expectations program. Couples were not eligible if either partner had previously participated in the Family Expectations program. All participants lived in or around Oklahoma City in the south‐central United States.

Interested couples visited the program office for an enrollment appointment, which included a meeting with a staff member and completion of baseline surveys. At baseline, all participants completed the Information, Family Outcomes, Reporting, and Management (nFORM) survey developed by Mathematica for ACF (see Strong et al., 2020 for information on the current version of nFORM) as well as the External Program Evaluator Survey (EPES). Each partner completed these surveys independently using a tablet. In most cases, the partners completed the survey in separate rooms. Following the planned random assignment procedures, 60% were assigned to receive the Family Expectations program (*n* = 786 couples).

Online resource Supporting Information Table S1 summarizes equivalence analyses comparing treatment and control groups. Results indicated no mean differences at baseline between conditions in all study variables (e.g., couple, parenting, and individual outcomes) as well as nearly all demographic variables (e.g., income, marital status). The one exception was age, with men and women assigned to the treatment group being, on average, approximately 7 months older than men are and women in the control group.

Follow‐up data collection occurred at the end of program completion for treatment couples (approximately 3–4 months following baseline assessment) as well as long‐term follow‐up for treatment and control participants (approximately 12 months following baseline assessment). The study was designed to have individuals complete nFORM‐based measures at postprogram and EPES‐based measures at 12‐month follow‐up (see Online Resource Supporting Information Figure S1). Postprogram surveys were given at the final session of the program. Hence, those who completed the postprogram survey were also individuals who attended either the entire program or most of the program (i.e., nearly 95% of the sample included in this study attended all 12 workshop sessions [mean = 11.94; *SD* = 0.33]). Treatment couples not attending the final program session were contacted at a later date about completing the nFORM Exit survey. Twelve‐month follow‐up assessments occurred at the program office as part of a scheduled follow‐up visit; those who did not attend the in‐person visit were invited to complete the survey online using a link sent by email or on a paper copy sent by mail. Each participant was paid $50 for completing the baseline survey and the 12‐month follow‐up survey.

The sample for this study comprised individuals from the treatment condition who completed the postprogram measures within 6 months of completion of the baseline survey (*N* = 676 individuals from 339 couples [two individuals lacked a partner completing the wave 2 survey]). Mean time from baseline to postprogram follow‐up was 3.75 months (*SD* = 0.85). Analyses comparing treatment couples included in the analytic sample versus treatment couples not included indicated the retained sample were older, more likely to be married, and had fewer biological children. In addition, women in the analytic sample reported lower levels of psychological distress than women in the treatment condition not included in the analytic sample. No group differences were observed with respect to couple functioning variables at baseline (e.g., destructive communication, relationship satisfaction).

Of the couples in the sample for this study, 91% of couples were living together, 39% were married, and 29% already had a child together (not considering the current pregnancy/newborn). Mean relationship length was 3.43 years (*SD* = 2.92). Average age of the participants at baseline was 27.23 years (*SD* = 6.20). The sample was racially diverse, with 55% being White, 28% Black/African American, 12% Native American, 3% Asian, 1% Native Hawaiian or other Pacific Islander, and 14% identified as a race not listed. In terms of ethnicity, 20% of the sample was Hispanic/Latino/a. A total of 67% had a high school diploma, and 67% were employed at least 20 h per week. The median combined income was in the $10,000–14,999 range.

The study was approved from the Institutional Review Board at *redacted* (study title: *redacted*; institutional review board approval number: *redacted*) and was not preregistered.

### Program delivery

Couples assigned to the intervention group were scheduled to begin attending Family Expectations workshops according to the couple′s availability. Family Expectations is a 12‐week program that delivers a 36‐h evidence‐based curriculum in workshop‐based sessions. The curriculum for Family Expectations, the Becoming Parents Program (Frei & Jordan, 2016), includes content focused on birth, transition to parenting together, stress management, caring for a newborn, strengthening the parenting relationship, as well as content adapted from the Prevention and Relationship Education Program for the couple relationship (PREP; e.g., Markman et al., 2010). Thus, Family Expectations can be conceived as an integrative program approach that incorporates practices observed in couple/relationship education as well as parenting education programming. The Family Expectations program underwent some changes since the previous randomized trials (i.e., Building Strong Families, Supporting Healthy Marriage); these changes include a reduction in case management from 12 to 15 meetings to meetings only as needed, and an increase in curriculum content from 30 to 36 h.

Workshop sessions occurred weekly for 12 weeks, with each session lasting 3 h. Couples were encouraged to arrive early to eat dinner, with the meal provided on‐site and at no cost to the couples. Childcare was also provided for their baby. Couples received monetary compensation for attendance at sessions, transportation, and childcare. As noted in the prior section, nearly 95% of the sample included in this study attended all 12 workshop sessions (mean = 11.94; *SD* = 0.33).

### Measures

The following 10 items were assessed on the nFORM assessment. Unless noted, nFORM items and measures were developed for this form by ACF and its subcontracted agencies.

### Couple outcomes (nFORM)

*Destructive Communication* in the past month was assessed using seven items. A sample item was “*My partner/spouse was rude or mean to me when we disagreed.*” Response options ranged from 1 = *never* to 4 = *often*. The measure demonstrated good reliability across waves 1 and 2 (*α* ≥ 0.89 for women and *α*≥ 0.89 for men). *Constructive Communication* in the past month was assessed using seven items. A sample item was “*My partner/spouse and I were good at working out our differences*.” Response options ranged from 1 = *never* to 4 = *often*. The measure demonstrated acceptable reliability across waves (*α* ≥ 0.61 for women and *α* ≥ 0.58 for men). *Emotional Support* within the relationship were assessed using 5 items. A sample item was “*I can count on my partner/spouse to be there for me.*” Response options ranged from 1 = *strongly agree* to 4 = *strongly disagree*. The measure demonstrated good reliability across waves (*α* ≥ 87 for women and *α* ≥ 0.88 for men). *Activities Together* during the past month was assessed using 3 items. A sample item included *“Talk to each other about the day.”* Response options ranged from 1 = *almost everyday* to 4 = *less often*. The measure demonstrated adequate reliability across waves (*α* ≥ 0.61 for women and *α* ≥ 0.53 for men). Items were reversed coded. *Relationship Satisfaction* was assessed using a single item that asked “*How satisfied are you with your current relationship.*” Response options include 1 = *very satisfied*, 2 = *somewhat satisfied*, and 3 = *not satisfied*. The item was reverse coded. *Relationship Longevity Beliefs* was assessed using one item that asked to which degree that participants agreed with the statement “*I view our marriage/relationship as lifelong*.” Response options ranged from 1 = *strongly agree* to 4 = *strongly disagree*. The item was reverse coded.

### Parenting outcomes (nFORM)

*Harsh Parenting* in the past month with their youngest child was assessed using two items. One item asked how often they “*Hit, spank, grab, or use physical punishment with [child′s name]*.” Response options include 1 = *never* to 4 = *often*. The measure demonstrated good reliability across waves (*α* ≥ 0.73 for women and *α* ≥ 0.60 for men). *Parenting Stress* in the past month was assessed using a single item question that “*how often have you felt overwhelmed by your parenting responsibilities?*” Response options ranged from 1 = *never* to 4 = *often*. *Coparenting Satisfaction* was assessed using a single item that asked participants to indicate their degree of agreement with the statement “*Child′s other parent and I work well together as parents*.” Answer options ranged from 1 = *strongly agree* to 4 = *strongly disagree*. The item was reverse coded.

### Individual outcomes (nFORM)

*Psychological distress* were assessed using 6 items from the Kessler Psychological Distress Scale (Kessler et al., 2002). A sample item from this scale asks respondents how frequently in the past 30 days they had felt that everything was an effort. Response options ranged from 1 = *none of the time* to 5 = *all of the time*. Internal consistency in the current sample was good across waves 1 and 2 (*α* ≥ 0.83 for women and *α* ≥ 0.84 for men).

Six EPES measures were included in this study, which are described subsequently.

### Couple outcomes (EPES)

*Relationship happiness* was assessed using 1 item that asked respondents to rate on a scale “*how happy would you say your relationship with partner is*.” Response options ranged from 0 = *not at all happy* to 10 = *completely happy*. *Partner Support* was assessed using 12 items from a measure employed in the Building Strong Families evaluation (McConnell et al., 2006). A sample item was “*partner listens to me when I need someone to talk to*.” Response options ranged from 1 = *strongly agree* to 4 = *strongly disagree*, which were then reverse coded. The measure demonstrated good reliability across waves 1 and 3 (*α* ≥ 0.91 for women and *α*≥ 0.91 for men). *Relationship Confidence* was assessed using 3 items from the Confidence Scale developed by Stanley et al. (1994). An example item was “*I believe we can handle whatever conflicts may arise in the future*”. Response options ranged from 1 = *strongly disagree* to 7 = *strongly agree*. The measure demonstrated good reliability across waves 1 and 3 (*α* ≥ 0.87 for women and *α* ≥ 0.92 for men). Multi‐item measures were averaged, with higher scores indicating higher confidence in relationship.

### Parenting outcomes (EPES)

*Coparenting* behaviors were assessed using 16 items from the Coparenting Relationship Scale developed by Feinberg et al., 2012). The scale asks respondents about the way they work together with their partners as parents. A sample item was “*I believe partner is a good parent*.” Response options ranged from 0 = *strongly disagree* to 6 = *strongly agree*. The measure demonstrated good reliability across waves 1 and 3 (*α* ≥ 0.88 for women and *α* ≥ 0.87 for men). Some items were reverse coded and averaged so that higher scores indicate better coparenting behaviors.

### Individual outcomes (EPES)

*Depressive symptoms* were measured using 12 items from Center for Epidemiologic Studies Depression Scale (Radloff, 1977). This scale asked participants about their depressive feelings during the past week. A sample item was “*I was bothered by things that usually don′t bother me*.” Response options ranged from 0 = *rarely or none of the time* to 3 = *most or all of the time*. The measure demonstrated good reliability across waves 1 and 3 (*α* ≥ 0.86 for women and *α* ≥ 0.88 for men). *Anger* during the past week was assessed using 5 items from an adaptation of the NIH PROMIS measure bank (Pilkonis et al., 2011). A sample item was “*I was ready to explode*.” Response options ranged from 1 = *not at all to* 5 = *very much*. The measure demonstrated good reliability across waves 1 and 3 (*α* ≥ 0.87 for women and *α*  ≥ 0.88 for men).

All measures with multiple items were averaged with higher scores indicating greater endorsement of that construct.

### Plan of analysis

Analysis of variance (ANOVA), multivariate analysis of variance (MANOVA), and multiple regression were employed in the analysis of the data. To examine the degree of change over time in nFORM measures, separate MANOVA models were conducted for couple outcomes (with six dependent variables) and for parenting outcomes (with three dependent variables); an ANOVA model was conducted for individual outcomes given the single dependent measure for this domain (i.e., psychological distress).

In all ANOVA and MANOVA models, sex was treated as a within subjects factor to account for the interdependent nature of dyadic data and adhere to assumptions of ANOVA testing, consistent with other studies (e.g., Barton et al., 2014). Accordingly, each case consisted of couple‐level data (i.e., both male and female partners were entered on the same line), providing total degrees of freedom based on the total number of couples, rather than total number of individuals. For statistically significant MANOVA main or interaction effects, univariate ANOVAs along with post hoc tests were performed to identify specific significant differences. These analyses were conducted using IBM SPSS 28 (IBM, 2021).

Analyses were executed in three stages corresponding to the three motivating research questions. The first stage focused on examining main effects of change over time and involved MANOVA and ANOVA models using a 2 (time) × 2 (sex) design, with time and sex as within‐subject factors. As no individuals in the current sample completed the nFORM survey at 12‐month follow‐up, analyses with 3 time assessments (i.e., a 3 × 2 design) were not possible.

The second stage involved multiple regression analyses to investigate the effects of short‐term change scores in key mechanisms targeted by the program (i.e., destructive and constructive communication, beliefs about relationship longevity, individual psychological well‐being) on 1‐year outcomes, controlling for baseline levels. These analyses permitted investigations into the degree to which change that occurred over the course of the program was predictive of more long‐term change in outcomes in the three domains of interest for this study, namely relationship (i.e., relationship happiness, partner support, relationship confidence), individual (i.e., depressive symptoms, anger), and parenting (i.e., coparenting). In all multiple regression equations, sex, age, education, marital status, and couple income were included as controls. As there were no sex‐specific hypotheses, analyses were executed at the individual level (i.e., “long” format), with individuals nested within dyads to account for the interdependence between partners using the CLUSTER command in Mplus version 8.3 (Muthén & Muthén, 2017).

The third stage focused on examining moderation effects between married and unmarried participants. This involved testing MANOVA and ANOVA models using a 2 (relationship status) × 2 (time assessment) × 2 (sex) design. In this model, time and sex were again treated as within‐subject factors and relationship status was a between‐subjects factor. Missing data was minimal (approximately 2% of data for waves 1 and 2 for analyses in SPSS and approximately 3% of data for waves 1–3 analyses in Mplus). Missing data was handled via Expectation‐Maximization in models ran with SPSS and full information maximum likelihood in models ran with Mplus.

## RESULTS

### Research Question 1: Change from preprogram to postprogram

Results of analyses examining change in mean levels from pre‐ to postprogram are summarized briefly in Table 1, with means and standard deviations reported in more detail in Online Resource Supporting Information Table S2. MANOVA analyses revealed a significant main effect of time for couple outcomes (*F*_(6, 339)_ = 10.87, *p* < 0.01) and parenting outcomes (*F*_(3, 151)_ = 11.54, *p* < 0.01). A significant main effect on time also appeared in ANOVA analyses for psychological distress (*F*_(1, 339)_ = 5.20, *p* < 0.05). These effects suggested general improvement over time and were subsequently investigated with univariate analyses.

**Table 1:** Summary of ANOVA and MANOVA models examining mean level pre‐post change in nFORM variables

	Model 1						Model 2			Model 3
	Destructive Comm.	Constructive Comm.	Emotional Support	Activities Together	R′ship Satis.	R′ship Long.	Harsh Parenting	Parent. Stress	Copar. Satis	Psyc. Distress
Time	−**	+**	+**	ns	+**	+**	−**	ns	+**	−*
Sex	ns	ns	**	**	**	**	**	**	**	ns
Time × sex	*	ns	*	ns	ns	ns	ns	ns	**	ns

	Model 4						Model 5			Model 6
Time	−**	+**	+**	ns	+**	+**	−**	ns	+**	−*
Sex	ns	ns	**	**	**	**	**	**	**	ns
Time × sex	ns	ns	ns	ns	ns	ns	ns	ns	**	ns
Time × marital status	*	*	ns	ns	ns	ns	*	ns	ns	ns
Time × sex × marital status	ns	ns	ns	ns	ns	ns	ns	ns	ns	ns

*Note*: Models 1–3 involving 2 (time) × 2 (sex) design. Models 4–6 involve 2 (time) × 2 (sex) × 2 (marital status) design. “+” indicates significant positive mean change over time (i.e., increase). “−” indicates significant negative mean change over time (i.e., decrease). *N* = 339 couples for Models 1 and 4; *N* = 151 couples for Models 2 and 5; *N* = 339 couples for Models 3 and 6. See Supporting Information Table S2 for means and standard deviations information.

Abbreviations: ANOVA, analysis of variance; MANOVA, multivariate analysis of variance; ns, nonsignificant.

* *p* < 0.05

** *p* < 0.01.

For couple outcomes, univariate analyses for the time main effect revealed significant effects in five of the six measures. Specifically, significant mean change was evident from pre‐ to postprogram for destructive communication (*F*_(1, 339)_ = 34.22, *p* < 0.01), constructive communication (*F*_(1, 339)_ = 41.94, *p* < 0.01), emotional support (*F*_(1, 339)_ = 17.02, *p* < 0.01), relationship satisfaction (*F*_(1, 339)_ = 17.40, *p* < 0.01), and relationship longevity beliefs (*F*_(1, 339)_ = 9.38, *p* < 0.01). In each instance, participants reported improved levels at postprogram compared to preprogram (e.g., higher constructive and lower destructive communication).

For parenting outcomes, univariate analyses for the time main effect revealed significant effects in two of the three dependent measures. In this, participants reported lower levels of harsh parenting (*F*_(1, 151)_ = 16.75, *p* < 0.01) as well as higher levels of coparenting satisfaction (*F*_(1, 151)_ = 17.78, *p* < 0.01) at postprogram compared to preprogram.

For the individual outcome measure, postprogram levels of psychological distress were significantly lower than preprogram levels (*F*_(1, 339)_ = 5.20, *p* < 0.05).

### Research Question 2: Short‐term change predicting long‐term change

To examine whether short‐term changes were associated with any subsequent change at 1‐year follow‐up, multiple regression equations were run. In this, change scores from pre to postprogram were computed for destructive communication, constructive communication, beliefs in relationship longevity, and psychological distress. The effects of change scores were investigated on couple, individual, and coparenting domains at 12‐month follow‐up.

Results of these analyses are summarized in Online Resource Supporting Information Table S3. Controlling for baseline levels, results indicated that changes in psychological distress were predictive of every outcome at the 1‐year follow‐up. Effects were in the expected direction; for instance, more declines in psychological distress from pre‐ to postprogram were associated with more increases in relationship happiness and more declines in depressive symptoms at 1‐year follow‐up controlling for baseline levels of those variables.

Changes in destructive communication were associated with five different outcomes at 1‐year follow‐up (relationship happiness, partner support, confidence, anger, and coparenting); all of these were significant and in the same direction as changes in psychological distress. Beliefs about relationship longevity were associated with changes at 1‐year follow up in four outcomes, being positively associated with changes relationship happiness, support, confidence, and negatively associated with changes in anger. Pre‐post changes in constructive communication were positively associated with changes in relationship happiness, perceived partner support, and coparenting.

As a follow‐up analysis, we ran regression models including short‐term change scores of all four nFORM measures for each outcome. Results of these analyses are summarized in Table 2. Controlling for the presence of change in other variables, psychological distress continued to demonstrate significant effects for changes in partner support, confidence, depressive symptoms, and anger. More positive change in beliefs about relationship longevity continued to be associated with relationship happiness and partner support, controlling for other variables. Changes in constructive communication and destructive communication only continued to demonstrate significant effects with partner support.

**Table 2:** Multivariate pre‐post change scores predicting change in outcomes at 1‐year follow‐up

	Relationship Happiness		Partner Support		Relationship Confidence		Depressive Symptoms			Anger		Coparenting	
	*B* (*β*)	*SE*	*B* (*β*)	*SE*	*B* (*β*)	*SE*	*B* (*β*)		*SE*	*B* (*β*)	*SE*	*B* (*β*)	*SE*
Δ_Pre‐Post_ CC	0.00 (0.01)	0.03	0.02 (0.09)*	0.01	0.02 (0.01)	0.05	0.04 (0.02)		0.11	0.01 (0.04)	0.01	0.04 (0.17)*	0.02
Δ_Pre‐Post_ DC	−0.02 (−0.05)	0.02	−0.01 (−0.08)*	0.01	−0.03 (−0.03)	0.03	−0.05 (−0.03)		0.09	0.01 (0.03)	0.01	−0.02 (−0.08)	0.02
Δ_Pre‐Post_ PD	−0.04 (−0.08)	0.02	−0.01 (−0.09)*	0.01	−0.06 (−0.07)	0.04	0.22 (0.13)*		0.09	0.02 (0.12)*	0.01	−0.02 (−0.08)	0.02
Δ_Pre‐Post_ RL	0.34 (0.09)	0.19	0.13 (0.12)*	0.05	0.86 (0.12)*	0.42	−0.64 (−0.05)		0.65	−0.08 (−0.06)	0.07	0.00 (0.00)	0.15
DV_Preprogram_	0.63 (0.53)**	0.05	0.82 (0.68)**	0.06	0.63 (0.51)**	0.07	0.55 (0.46)**		0.06	0.44 (0.43)**	0.05	0.47 (0.44)**	0.10
Age	0.02 (0.05)	0.01	0.00 (0.01)	0.00	0.02 (0.03)	0.02	−0.08 (−0.07)		0.06	−0.01 (−0.09)*	0.01	0.02 (0.12)	0.01
Education	0.04 (0.04)	0.05	0.01 (0.04)	0.01	0.14 (0.07)	0.09	−0.00 (−0.00)		0.18	−0.02 (−0.03)	0.02	0.05 (0.09)	0.04
Sex	−0.49 (−0.12)**	0.13	−0.09 (−0.08)**	0.03	−0.56 (−0.07)*	0.26	0.38 (0.03)		0.51	0.04 (0.02)	0.06	−0.17 (−0.09)	0.12
Married	0.16 (0.04)	0.17	0.06 (0.05)	0.04	0.46 (0.06)	0.31	−1.15 (−0.07)		0.68	−0.16 (−0.09)*	0.07	0.07 (0.04)	0.16
Income	−0.04 (−0.09)	0.02	−0.01 (−0.06)	0.01	−0.03 (−0.04)	0.03	0.02 (0.02)		0.07	0.00 (0.02)	0.01	−0.02 (−0.11)	0.02

*Note*: Analytic sample sizes given missing data patterns were as follows: 568 (Relationship Happiness), 572 (Partner Support), 571 (Relationship Confidence), 619 (Depressive Symptoms), 623 (Anger), and 176 (Coparenting).

Abbreviations: CC, constructive communication; DC, destructive communication; DV, dependent variable; PD, psychological distress; RL, relationship longevity.

* *p* < 0.05;

** *p* < 0.01.

### Research Question 3: Moderation by marital status

**Figure 1:**
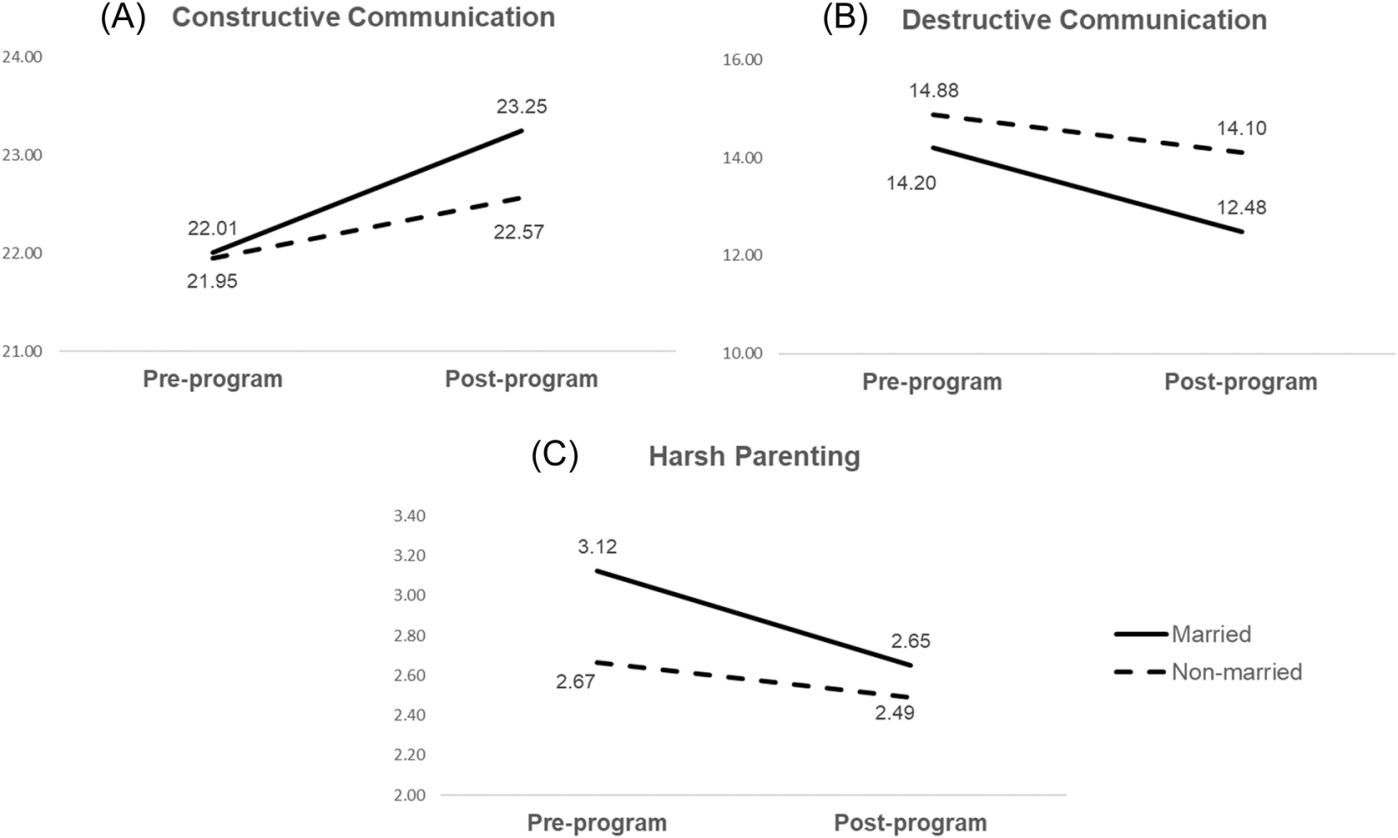
Plotting pre/post mean levels for significant time × marital status interaction

Results of 2 × 2 × 2 MANOVAs and ANOVA models are summarized in Table 1 and Figure 1. Consistent with the third research question, attention is focused on the time × marital status interaction. Findings indicated three measures with a significant time × marital status interaction: constructive communication, destructive communication, and harsh parenting.

To more clearly illustrate these interactions, we plotted mean levels of each measure by couple type. For constructive communication (Figure 1A) and destructive communication (Figure 1B), mean score comparisons revealed married individuals exhibited greater positive change relative to the change reported by unmarried individuals. This significant time × marital status also qualifies the overall time main effect on these measures, suggesting that overall changes in constructive and destructive communication were more attributable to changes observed in married individuals than unmarried. With respect to harsh parenting, results indicated that married individuals reported more declines in harsh parenting relative to unmarried individuals (Figure 1C).

For the remaining outcomes, the time × marital status interaction was nonsignificant, indicating married and unmarried respondents did not differ in mean levels of change over time. In addition, the three‐way interaction of time × sex × marital status was also not significant in all models.

## DISCUSSION

Results from programming funded by federal initiatives to support healthy couple relationships have been varied and, for the largest‐scale studies, rather muted. Although some have viewed these findings and suggested the abandoning of the efforts (Karney et al., 2018), the empirical body of research on the significance of healthy stable couple relationships for the well‐being of adults, children, and communities would seem to suggest continued warrant for efforts in this area, albeit perhaps with new types of programming or tailored delivery strategies. As described below, results from the current study help to inform this ongoing debate by demonstrating the nature of change in couple, parenting, and individual domains following participation in the Family Expectations program for married and unmarried couples.

To begin, results from the study indicated significant mean‐level improvements shortly following program participation in couple, parenting, and individual domains among this subsample of participants who attended most or all of the program sessions. That improvements were observed in all these domains is particularly notable given that the program was targeted to new or expectant parents – a time when relationship quality tends to decline (Doss & Rhoades, 2017). In addition, these findings are consistent with some of the original rationale for this type of funding, namely that targeting couple relationships may promote functioning across couple‐ and noncouple‐specific domains. The lack of control group data at short‐term follow‐up limits our ability to isolate change strictly due to program participation, but these findings do document change occurring at a time when, as noted above, relationship quality tends to deteriorate. Relatedly, these findings suggest the potential for some program effects at postprogram in more domains than were observed at 1‐year follow‐up (cf. Ritchie et al., 2022). If so, this would suggest that program effects were occurring initially within couples, but were not sustained (apart from improvements in destructive communication for the entire intervention group relative to controls). However, as we note in the limitations section, those completing the post assessment tended to be couples who completed most or all of the intervention. As such, these findings speak most clearly to the initial gains and maintenance of gains on a number of outcomes within that group.

Additional analyses indicated change shortly following the program had prognostic significance for change in outcomes at the 1‐year follow‐up. Although we are unable to document causality, these findings nevertheless show that change from pre‐ to postprogram was meaningfully related to more long‐term change scores in similar constructs. This finding is also consistent with prior research linking short‐term change in relationship functioning following participation in relationship education programming with change in more long‐term outcomes in both adults (Barton et al., 2021) and children (Lavner et al., 2020).

Among the variables examined in the current study, short‐term change in psychological distress demonstrated significant effects on the most number of long‐term outcomes, even after accounting for change scores in other measures. Thus, in addition to results suggesting that participation may foster positive change in individuals' mental health over the course of the program, this second set of analyses indicated this change in psychological distress (more so than changes in couple measures) was associated with change in a wide variety of outcomes more than 6 months later. This collateral benefit from relationship education for individuals' mental health is further supported in other prevention programming (Roddy et al., 2020) as well as clinical practice (Beach et al., 1991). These findings are also important because psychological distress during this period (pregnancy and after the birth of a baby) may be reflective of postpartum depression. Future research measuring postpartum depression directly, as well as program effects on this outcome, would be valuable.

The Family Expectations program provides an integrative model of programming for new and expectant parents that includes elements of both couple/relationship and parenting education. Our findings are consistent with this model, with change noted across multiple domains. As noted in the preceding paragraph, short‐term change in psychological distress demonstrated the most consistent associations with long‐term change in multiple outcomes, highlighting the value of providing individuals with programming that can reduce levels of psychological stress, at least during this unique time around the birth of baby. Identifying which particular aspect(s) of the curriculum may have been most influential for change in psychological distress is beyond the scope of the present study but provides an important question for future research as a means to optimize program content and delivery. Overall, the integration (and separation) of relationship education and mental health services remains important area for further scientific inquiry.

Analyses investigating the effect of marital status were generally nonsignificant, indicating mostly no differences in short‐term change between married and unmarried participants. In instances when the moderation was significant, a consistent pattern emerged with married couples demonstrating greater improvements relative to unmarried participants. This suggests that while “at risk” couples, as defined according to sociodemographic variables (e.g., education, income), have been found to benefit more from relationship education programming (Stanley et al., 2020), this pattern may not translate to risk status defined by relationship type. That is, married couples—those who have already made a public, formal commitment to the longevity of the relationship—appeared to benefit more for some certain aspects of the relationship and parenting education program compared to those that had not done so. Given the sample size for these analyses (particularly for parenting variables), we limit the overall conclusions that can be drawn from null findings and highlight the need for future research with more powered samples that investigates differences by relationship type.

Various limitations of the study should be acknowledged when interpreting its findings. As previously mentioned, no data were collected from control participants at postprogram with these measures. Thus, current findings cannot empirically document if observed changes were exclusively due to treatment assignment. Second, given the design for postprogram data collection, the sample comprised treatment couples that generally attended all (or nearly all) of the program. Thus, findings should be interpreted as occurring with respect to changes following participation (and not relative to a control condition) within a sample of couples that attended most or all of the intervention. Third, attrition analyses indicated the participating couples that completed postprogram data were older, more educated, and had women with lower psychological distress than treatment couples without such data; that said, the direction of these differences suggests current findings may underestimate effects given increased benefits that have been documented with more socioeconomically at‐risk couples. Fourth, sample size limitations precluded the ability to infer strong conclusions of null findings, particularly with parenting variables. Continued innovative measurement approaches are needed when working with couples expecting the birth of their first child to understand meaningful change in parenting outcomes. Fifth, the study required dyadic participation, potentially resulting in a sample with higher levels of relationship functioning than the general population (see Barton et al., 2020a; 2020b). Finally, given enrollment criteria, caution is warranted in overgeneralizing findings to other populations receiving this type of relationship education not also expecting the birth of a baby.

To conclude, we highlight three specific implications for research and programming in this area. First, continued work is needed strengthen the sustained impact of programming. Although short‐term changes were observed in the current study, other research from this RCT suggested more limited long‐term effects (Ritchie et al., 2022). Second, results indicate the importance of developing and implementing programs that couples attend. As attendance rates of this program and other programs funded by this ACF initiative attest, couple attendance in programming varies dramatically. Third, important questions remain for what relationship education should entail across the spectrum of couple relationship types that participate. Should programming be equivalent for couples that have already stated their intent for a lifelong relationship together versus those who are still trying to decide this? The number and nature of developmental tasks different couples are attempting to accomplish (e.g., maintain a supportive relationship; decide future and mutual commitment to the relationship; becoming parents) merits consideration as a means to inform the next generation of innovative and effective programs for new and expectant parents.

## Supporting information

Supporting information.
