# Supplementary material for: Changes in couple, parenting, and individual functioning following Family Expectations program participation
Source: J Marital Fam Ther. 2022 Sep 24;49(1):169–85. doi: 10.1111/jmft.12613 (PMC10086790; doi:10.1111/jmft.12613)
Supplement: Supplementary file 1 — Supporting information. [file JMFT-49-169-s001.docx]

**Changes in couple, parenting, and individual functioning following Family Expectations program participation**

**Supplemental Material**

Supplemental Table 1. Equivalence Analyses of Treatment and Control Conditions at Baseline

|  | Treatment (n=786 couples) | | | |  | | Control (n=534 couples) | | | |  | Test Statistic ^b^ | | |  |
| --- | --- | --- | --- | --- | --- | --- | --- | --- | --- | --- | --- | --- | --- | --- | --- |
|  | Women | | Men | |  | | Women | | Men | |  | Women | Men | |  |
| Couple outcomes | |  | |  | |  | |  | |  | |  | |  | |
| Destructive Communication | 15.02 (5.38) | | 14.92 (5.20) | |  | | 15.37 (5.52) | | 15.28 (5.32) | |  | 1.153 | 1.211 | |  |
| Constructive Communication | 21.84 (3.34) | | 21.92 (3.19) | |  | | 21.61 (3.36) | | 21.65 (3.20) | |  | -1.248 | -1.491 | |  |
| Emotional Support | 16.81 (3.06) | | 17.40 (2.84) | |  | | 16.69 (3.04) | | 17.31 (2.94) | |  | -.738 | -.532 | |  |
| Activities Together | 10.80 (1.63) | | 11.05 (1.37) | |  | | 10.69 (1.63) | | 10.97 (1.55) | |  | -1.226 | -.889 | |  |
| Relationship Satisfaction | 2.61 (0.56) | | 2.72 (0.49) | |  | | 2.58 (.56) | | 2.67 (.53) | |  | -.840 | -1.838 | |  |
| Relationship Longevity | 3.61 (0.60) | | 3.65 (0.59) | |  | | 3.61 (.58) | | 3.64 (.57) | |  | .136 | -.357 | |  |
|  |  | |  | |  | |  | |  | |  |  |  | |  |
| Parenting Outcomes | |  | |  | |  | |  | |  | |  | |  | |
| Harsh Parenting | 2.82 (1.24) | | 2.58 (1.04) | |  | | 2.71 (1.16) | | 2.66 (1.21) | |  | -1.036 | .755 | |  |
| Parenting Stress | 2.44 (0.89) | | 2.06 (0.94) | |  | | 2.43 (0.91) | | 2.08 (0.94) | |  | -.155 | .236 | |  |
| Coparenting Satisfaction | 3.38 (0.70) | | 3.63 (0.56) | |  | | 3.39 (0.65) | | 3.64 (0.52) | |  | .137 | .267 | |  |
|  |  | |  | |  | |  | |  | |  |  |  | |  |
| Individual Outcome | |  | |  | |  | |  | |  | |  | |  | |
| Psychological Distress | 12.43 (4.82) | | 11.46 (4.71) | |  | | 12.61 (4.97) | | 11.66 (4.70) | |  | .654 | .765 | |  |
| Demographic Variables |  | |  | |  | |  | |  | |  |  |  | |  |
| Age | 25.35 (5.20) | | 27.56 (6.74) | |  | | 24.74 (4.86) | | 26.80 (6.33) | |  | -2.146* | -2.043* | |  |
| Education | 3.75 (1.81) | | 3.41 (1.84) | |  | | 3.74 (1.76) | | 3.39 (1.82) | |  | -.060 | -.196 | |  |
| Child Together ^a^ | 0.31 | | --- | |  | | 0.30 | | --- | |  | .426 | --- | |  |
| Married ^a^ | 0.30 | | --- | |  | | 0.28 | | --- | |  | .867 | --- | |  |
| Income ^a^ | 6.83 (5.11) | | --- | |  | | 6.62 (5.04) | | --- | |  | -.733 | --- | |  |

Note: ^a^ couple level variables (determined by male and female partner reports). ^b^ *T*-test for continuous and ordinal variables; chi-square cross-tab statistic for binary variables. Standard deviations not reported for binary variables. * *p* < .05.

Supplemental Table 2. Means (Standard Deviations) for Multivariate 2 × 2 Analyses

|  | Pre-Program | |  | Post-Program | |
| --- | --- | --- | --- | --- | --- |
|  | Women | Men |  | Women | Men |
| MANOVA (Couple outcomes; n=339 couples) | | |  |  |  |
| Destructive Communication | 14.64 (5.34) | 14.59 (5.08) |  | 13.17 (4.74) | 13.78 (4.81) |
| Constructive Communication | 21.81 (3.47) | 22.14 (2.99) |  | 22.88 (2.86) | 22.78 (2.81) |
| Emotional Support | 16.94 (2.95) | 17.69 (2.71) |  | 17.58 (2.86) | 18.00 (2.41) |
| Activities Together | 10.77 (1.53) | 11.10 (1.22) |  | 10.76 (1.49) | 11.09 (1.20) |
| Relationship Satisfaction | 2.64 (0.52) | 2.75 (0.48) |  | 2.75 (0.48) | 2.82 (0.41) |
| Relationship Longevity | 3.64 (0.56) | 3.73 (0.49) |  | 3.72 (0.57) | 3.79 (0.45) |
|  |  |  |  |  |  |
| MANOVA (Parenting Outcomes; n = 151 couples) | | |  |  |  |
| Harsh Parenting | 2.96 (1.31) | 2.71 (0.92) |  | 2.64 (1.06) | 2.46 (0.82) |
| Parenting Stress | 2.43 (0.86) | 2.11 (0.80) |  | 2.46 (0.88) | 2.00 (0.79) |
| Coparenting Satisfaction | 3.26 (0.66) | 3.64 (0.44) |  | 3.50 (0.56) | 3.69 (0.50) |
|  |  |  |  |  |  |
| ANOVA (Individual Outcome; n = 339 couples) | | |  |  |  |
| Psychological Distress | 11.90 (4.43) | 11.15 (4.25) |  | 11.21 (4.65) | 11.00 (4.51) |

Supplemental Table 3. Pre-post change score predicting changes in outcomes at 1-year follow-up

|  | Relationship Happiness | Support | Confidence | Depressive Symptoms | Anger | Co-parenting |
| --- | --- | --- | --- | --- | --- | --- |
| Models w/ DC | Model 1 | Model 2 | Model 3 | Model 4 | Model 5 | Model 6 |
| Δ_Pre-Post_ DC | -.05 (.02)** | -.05(.02)** | -.08(.03)* | .05(.07) | .02(.01) | -.03(.01)* |
| Baseline DV | .62(.06)** | .62(.06)** | .61(.07)** | .51(.06)** | .42(.05)** | .40(.09)** |
| Age | .02 (.01) | .02(.01) | .02(.03) | -.09(.06) | -.01(.01)* | .02(.01)* |
| Education | .03 (.05) | .03(.05) | .11(.09) | .05(.18) | -.01(.02) | .05 (.04) |
| Sex | -.48 (.13)** | -.48(.13)** | -.51(.27) | .34(.50) | .03(.06) | -.13(.12) |
| Married | .17 (.17) | .17(.17) | .55(.33) | -1.24(.70) | -.17(.07)* | .07 (.15) |
| Income | -.04 (.02)* | -.04(.02)* | -.03(.03) | .03(.07) | .01(.01) | -.02(.02) |
| Models w/ CC | Model 7 | Model 8 | Model 9 | Model 10 | Model 11 | Model 12 |
| Δ_Pre-Post_ CC | -.00(.04) | .03(.01)** | .09(.05) | -.02(.10) | -.00(.01) | .05(.02)** |
| Baseline DV | .41(.05)** | .76(.06)** | .61(.07)** | .51(.06)** | .41(.05)** | .43(.09)** |
| Age | -.10(.04)* | .00(.00) | .03(.03) | -.09(.06) | -.01(.01)* | .02(.01)* |
| Education | -.02(.04) | .01(.01) | .11(.09) | .05(.18) | -.01(.02) | .05(.04) |
| Sex | .02(.03) | -.08(.03)* | -.50(.27) | .34(.50) | .03(.06) | -.16(.11) |
| Married | -.10(.04)* | .07(.05) | .52(.32) | -1.25(.70) | -.17(.07)* | .05(.14) |
| Income | .02(.04) | -.01(.01) | -.03(.03) | .03(.07) | .00(.01) | -.02(.02) |
| Models w/ RL | Model 13 | Model 14 | Model 15 | Model 16 | Model 17 | Model 18 |
| Δ_Pre-Post_ RL | .12(.05)* | .19(.05)** | 1.03(.40)* | -.75(.62) | -.12(.07) | .07(.15) |
| Baseline DV | .51(.04)** | .74(.06)** | .60(.07)** | .51(.06)** | .41(.05)** | .35(.09)** |
| Age | .06(.04) | .00(.00) | .03(.02) | -.09(.06) | -.01(.01)* | .02(.01)* |
| Education | .03(.04) | .01(.01) | .13(.09) | .04(.18) | -.01(.02) | .05(.04) |
| Sex | -.11(.03)** | -.08(.03)* | -.54(.27)* | .34(.51) | .03(.06) | -.13(.12) |
| Married | .04(.04) | .08(.05) | .51(.32) | -1.24(.69) | -.17(.07)* | .07(.16) |
| Income | -.09(.05) | -.01(.01) | -.03(.03) | .03(.07) | .00(.01) | -.01(.02) |
| Models w/ PD | Model 19 | Model 20 | Model 21 | Model 22 | Model 23 | Model 24 |
| Δ_Pre-Post_ PD | -.05(.02)* | -.02(.01)** | -.10(.04)** | .24(.08)** | .03(.01)** | -.03(.02) |
| Baseline DV | .60(.05)** | .73(.06)** | .61(.07)** | .54(.06)** | .45(.05)** | .38(.09)** |
| Age | .02(.01) | .00(.00) | .02(.02) | -.08(.05) | -.01(.01)* | .01(.01) |
| Education | .04(.04) | .02(.01) | .13(.09) | -.04(.17) | -.02(.02) | .06(.04) |
| Sex | -.48(.13)** | -.09(.03)* | -.54(.27)* | .28(.51) | .03(.06) | -.16(.12) |
| Married | .19(.17) | .07(.05) | .54(.32) | -1.10(.66) | -.16(.07)* | .08(.14) |
| Income | -.03(.02) | -.01(.01) | -.03(.03) | .02(.07) | .00(.01) | -.02(.02) |

**Notes: *** *p* < . 05; ****** *p* < . 01. DC = Destructive Communication. CC = Constructive Communication. RL = Relationship Longevity. PD = Psychological Distress. DV = Dependent Variable.

Supplemental Figure 1. Summary of Data Collection

EPES &

nFORM

completed (FE and control)

FE Program Participation (FE only)

nFORM completed (FE subsample)

EPES completed (FE and Control)

Pre-program (baseline)

Post-program

(~3-6 mo. post baseline)

Long-term follow-up

(~12 mo. post baseline)

Notes: EPES = External Program Evaluator Survey. nFORM = Information, Family Outcomes, Reporting, and Management. FE = Family Expectations
